# Neuroinflammatory Markers in the Serum of Prepubertal Children with Down Syndrome

**DOI:** 10.1155/2020/6937154

**Published:** 2020-03-23

**Authors:** Luigi Tarani, Valentina Carito, Giampiero Ferraguti, Carla Petrella, Antonio Greco, Massimo Ralli, Marisa Patrizia Messina, Debora Rasio, Enrica De Luca, Carolina Putotto, Paolo Versacci, Mauro Ceccanti, Marco Fiore

**Affiliations:** ^1^Department of Pediatrics, Sapienza University Hospital of Rome, Italy; ^2^Institute of Biochemistry and Cell Biology, Section of Neurobiology, National Research Council (IBBC-CNR), Rome, Italy; ^3^Department of Cellular Biotechnologies and Hematology, Sapienza University Hospital of Rome, Italy; ^4^Department of Sense Organs, Sapienza University Hospital of Rome, Italy; ^5^Department of Obstetrics and Gynecology, Sapienza University Hospital of Rome, Italy; ^6^San Raffaele Roma Open University, Rome, Italy; ^7^Centro Riferimento Alcologico Regione Lazio, Sapienza University of Rome, Italy

## Abstract

Down Syndrome (DS) is the most common chromosomal disorder. Although DS individuals are mostly perceived as characterized by some distinct physical features, cognitive disabilities, and cardiac defects, they also show important dysregulations of immune functions. While critical information is available for adults with DS, little literature is available on the neuroinflammation in prepubertal DS children. We aimed to evaluate in prepubertal DS children the serum levels of nerve growth factor (NGF) and brain-derived neurotrophic factor (BDNF), oxidative stress as free oxygen radicals defense (FORD), free oxygen radicals test (FORT), and cytokines playing key roles in neuroinflammation and oxidative processes as TNF-*α*, TGF-*β*, MCP-1, IL-1*α*, IL-2, IL-6, IL-10, and IL-12. No differences were found in NGF between DS children and controls. However, BDNF was higher in DS subjects compared to controls. We also did not reveal changes in FORD and FORT. Quite interestingly, the serum of DS children disclosed a marked decrease in all analyzed cytokines with evident differences in serum cytokine presence between male and female DS children. In conclusion, the present study evidences in DS prepubertal children a disruption in the neurotrophins and immune system pathways.

## 1. Introduction

Down Syndrome (DS), also known as trisomy 21 for the presence of an extra copy of the chromosome 21 that leads to a wide spectrum of cognitive and physical symptoms, is one of the most common chromosomal disorders occurring in about 1 in 800 newborns worldwide [1]. The extra chromosome disrupts the normal course of physical development and results in mild to moderate intellectual disabilities. DS people are also characterized by some distinct physical features, such as a flat-looking face, and by a high risk for a number of other health conditions including infections, alteration of the immune system, thyroid, pulmonary, skeletal, skin, hearing and vision issues, diabetes, sleep apnea, seizures, early menopause, and congenital heart defects [2]. There are a number of studies showing changes in several aspects of the humoral and cellular immune system associated with DS, including functional impairments of B and T lymphocytes and natural killer cells and dysfunctions in phagocytosis and chemotaxis of polymorphonuclear leukocytes [3–5].

Individuals with DS exhibit disturbances in thymic development [4] and also show, besides low B-lymphocyte numbers, a dysregulated immunoglobulin pattern [6]. There are also a number of reports showing increased levels of cytokines leading to a proinflammatory profile, such as Interferon-*γ* (IFN-*γ*), Interleukin-6 (IL-6), and Tumor Necrosis Factor-alpha (TNF-*α*), in individuals with DS [7–9].

It is known that chromosome 21 of humans and chromosome 16 of mice carry genes that are involved in the function of the IFN family of cytokines and receptors [5] and that overexpression of chromosome 21-gene products causes changes in inflammatory cytokines in the blood [10], as TNF-*α* and IFN-*γ* that have several biological effects in the body, including important regulatory roles in immune responses. It has been hypothesized that abnormal production of proinflammatory cytokines might participate in the neuropathological changes associated with DS, such as Alzheimer's-like mental retardation [8, 11].

Studies on inflammatory and autoimmune diseases, which are characterized by abnormal activation of immune cells and an increased production of cytokines, have revealed a localized increase in NGF at the sites of inflammation or in the blood [12]. NGF plays an essential role in the differentiation, survival, and functions of neuronal cells in the central and peripheral nervous system [13, 14]. Moreover, NGF has a role outside the nervous system and in the cardiovascular and immune systems [15–17]. NGF expression and functional activities have now been well demonstrated for human basophils, monocyte/macrophages, and T- and B-lymphocytes [18, 19]. In this context, a number of studies have shown that the plasma and/or serum NGF levels are disrupted in several autoimmune, inflammatory, and fibrotic disorders [20–23]. Brain-derived neurotrophic factor (BDNF) is another growth factor which plays a central role in the survival and differentiation of neurons [24, 25], but apart from nervous system disorders, several reports documented an association between plasma BDNF and systemic or peripheral inflammatory conditions [26]. It has been shown that activated antigen-specific T cells, B cells, and monocytes produce BDNF and IL-6 [27–29]. Indeed, TNF-*α* represents a specific link between monocyte infiltration and neuronal changes in inflammatory diseases. Furthermore, TNF-*α* and IL-6 play an important role in regulating BDNF secretion [27–29].

Several evidences suggest a significant increase in infectious and autoimmune diseases in individuals with DS, independent of gender, age, family history, and exposure to other risk factors, suggesting an intrinsic alteration of the immune system [30]. Since DS is known to be characterized by a proinflammatory profile and then by an increase in proinflammatory cytokines, including TNF-*α* and IL-6, it is reasonable to hypothesize a concomitant alteration in the circulating levels of neurotrophins.

Oxidative stress is a phenomenon associated with an imbalance between the production of free radicals and reactive metabolites (e.g., superoxide and hydrogen peroxide) and the antioxidant defenses. Oxidative stress has been associated with the known various morphological abnormalities, immune disorders, intellectual disability, premature aging, and other biochemical abnormalities of DS individuals; furthermore, the Cu/Zn superoxide dismutase (SOD) gene is located on the 21st chromosome [31]. Indeed, many studies examined the impact of antioxidant interventions as well as the positive effect of physical exercise on cognitive and learning disabilities of individuals with DS proposing BDNF as a potential therapeutic target at the molecular level [31–33].

Although crucial findings are available for adults with DS, only a few data are existing on the correlation between neuroinflammation and oxidative stress in prepubertal, the period of life immediately before puberty, DS children. Thus, the current study was designed to determine and correlate in prepubertal male and female DS children (i) the serum levels of NGF and BDNF; (ii) the oxidative status measured in the serum as free oxygen radicals defense (FORD) and free oxygen radicals test (FORT); and (iii) the serum levels of cytokines playing subtle roles in both neuroinflammatory and oxidative processes as TNF-*α*, transforming growth factor-beta (TGF-*β*), Monocyte Chemoattractant Protein-1 (MCP-1), IL-1*α*, IL-2, IL-6, IL-10, and IL-12.

## 2. Materials and Methods

### 2.1. Participants' Selection

Patients enrolled in the present study were 9 individuals with Down Syndrome in follow up at the genetics ambulatory of the Department of Pediatrics of the Sapienza University Hospital “Policlinico Umberto I” of Rome, Italy. DS participants were 9 prepubertal children (5 males and 4 females) aged between 1 and 9.6 years who have been diagnosed for Down Syndrome with free trisomy 21 by both clinical and molecular analyses. The karyotype analysis involved different steps: first, the culture of lymphocytes, then the chromosome banding, and in the end a microscopic analysis [34]. For all patients, a peripheral venous blood sample was collected and conserved in 3 ml heparin tube from which the lymphocytes were isolated and cultured for 72 hours at 37°C [35, 36]. The chromosomal banding was then carried out using the G-banding (Giemsa banding using Trypsin) kit by Thermo Fisher (Waltham, MA, USA) performed according to the manufacturer's instructions [36]. For each patient blood sample, 100 metaphase cells with an average resolution of 400 bands were recorded. For DS children recruitment, several investigations were carried out: (1) physiological anamnesis: type of delivery and gestational age at the time of birth, type of breastfeeding, age of weaning, eating habits, and age; (2) close and remote pathological anamnesis; (3) pharmacological anamnesis; (4) family history: diseases, parents' age at pregnancy, and parents' education; and (5) physical examination and anthropometric parameters measurements: gender, ethnicity, weight, and height. In DS prepubertal children, the peripheral venous blood sample was collected for the karyotype analysis.

The control group consisted of 21 age-matched healthy prepubertal children (10 males and 11 females) selected at the Department of Pediatrics of the Sapienza University Hospital “Policlinico Umberto I” of Rome, Italy. We have selected these children because they were recovered in the hospital for the investigation of the presumed pathologies that resulted not present at all, defining thus the children “healthy.” For both control children and DS individuals, the peripheral venous blood sample was collected at the moment of discharge for routine analyses.

Main exclusion criteria for the enrollment in the study for all children included (1) puberty in place or postpuberty; (2) use of drugs or chemicals that can alter the serum levels of inflammation markers, such as antidepressants, anti-inflammatory, and immunosuppressant; (3) inflammatory, endocrine, and autoimmune disorders such as thyroiditis and celiac disease; (4) other ongoing pathologies; and (5) children with diagnosed cardiovascular pathologies that could have biased inflammatory analyses. We consider puberty as the process of physical changes through which a child's body matures into an adult body capable of sexual reproduction. We included the enrolled subjects in the prepubertal age phase by Tanner scale examination by pediatricians with expertise in sexual development. The study was approved by the Sapienza University Hospital ethical committee; an informed consent was signed by each parent of the children and all the study procedures were in accordance with the Helsinki Declaration of 1975, as revised in 1983, for human experimentation.

### 2.2. NGF and BDNF Serum Level Evaluation

Peripheral blood samples of 5 ml were taken from each participant, collected in heparinized tubes, and centrifuged at 3000 rpm for 15 min to separate serum from plasma. The serum separated from the blood was then stored at –80°C. NGF (Cat. No. DY256) and BDNF (Cat. No. DY248) were measured using a sandwich enzyme-linked-immunosorbent assay (ELISA) kits (R&D Systems, Minneapolis, MN, USA), according to the protocols provided by the manufacturer and also according to methods previously described [37–39]. Serum samples were diluted 2- and 100-fold with PBS for detection of NGF and BDNF, respectively. The colorimetric reaction product was measured at 450 nm using a microplate reader (Dynatech MR 5000, PBI International, USA). Data are represented as pg/mg total proteins and all assays were performed in duplicate which was averaged for statistical comparison.

### 2.3. Free Oxygen Radicals Defense (FORD) and Free Oxygen Radicals Test (FORT)

FORD and FORT tests were carried out using two specific kits (both purchased by Callegari, Parma, Italy) following the instruction provided by the manufacturer and according to methods previously described [37–39]. Blood serum was used both for the FORT and FORD determination. FORD test allows the determination of free oxygen radicals defense. Briefly, this test uses a preformed stable and colored radical and determines the decrease in absorbance that is proportional to the antioxidant concentration of the sample [40].

By contrast, the FORT test allows the determination of free oxygen radicals (ROS) through a colorimetric assay based on the ability of transition metals, such as iron, to catalyze the breakdown of hydroperoxides (ROOH) into derivative radicals, according to Fenton's reaction [40].

### 2.4. Oxidative Stress ELISA Strip Profiling Assay

Expression patterns of cytokines were studied in the enrolled DS prepubertal children and healthy control children by using Human Oxidative Stress ELISA Strip for Profiling 8 Cytokines (Catalog Number EA-1301, purchased by Signosis) that simultaneously analyzes the following cytokines: TNF-*α*, TGF-*β*, MCP-1, IL-1*α*, IL-2, IL-6, IL-10, and IL-12. Briefly, 100 *μ*l of the sample was added per well and was incubated for 2 hours at room temperature with gentle shaking. Then, each well was aspirated and washed, three times, by adding 200 *μ*l of 1X Assay wash buffer. After the last wash, 100 *μ*l of the diluted biotin-labeled antibody mixture was added to each well and incubated for 1 hour at room temperature, with gentle shaking. Then, the aspiration/wash was repeated as described above. 100 *μ*l of diluted streptavidin-HRP conjugate was added to each well and incubated for 45 min at room temperature with gentle shaking. The plate was washed again and 100 *μ*l substrate was added to each well and incubated for 10-30 minutes. 50 *μ*l of Stop solution was added to each well observing a color change from blue to yellow. Then, the optical density of each well was determined with a microplate reader at 450 nm within 30 minutes.

### 2.5. Statistical Analysis

Serum NGF, BDNF, FORT/FORD, and cytokines in DS prepubertal children were analyzed using two-way analysis of variance (ANOVA) (DS children versus control children and gender effect). Post hoc comparisons were performed using Tukey's HSD test.

## 3. Results

### 3.1. Characteristics of the Subjects Included in the Study

Table 1 shows the anamnestic and anthropometric findings of the children and their parents included in the study. No differences between groups were revealed by statistical analyses because data on age, weight, height, and BMI displayed high variability between children.

### 3.2. NGF and BDNF Serum Levels

**Figure 1:**
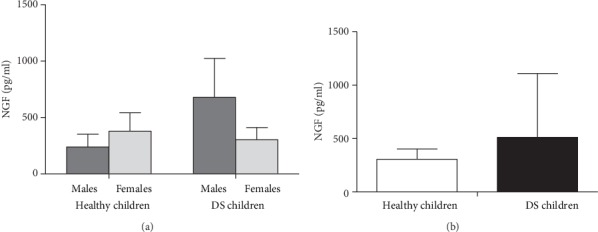
Nerve growth factor (NGF) levels in male and female DS prepubertal children and in the control group ((a) shows the data according to gender but (b) are without it). The error bars indicate pooled standard error means (SEM) derived from appropriate error mean square in the ANOVA.

**Figure 2:**
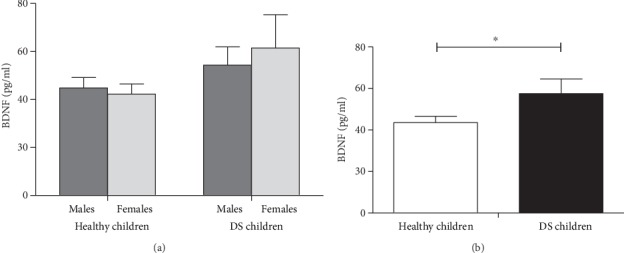
Brain-derived neurotrophic factor (BDNF) levels in male and female DS prepubertal children and in the control group ((a) shows the data according to gender but (b) are without it). The error bars indicate pooled standard error means (SEM) derived from appropriate error mean square in the ANOVA. The asterisk indicates significant differences between groups (^∗^*p* < 0.05).

Figures 1 and 2 show the serum levels of NGF and BDNF, respectively, in male and female DS prepubertal children and in the control group (Figures 1(a) and 2(a) show the data according to gender but Figures 1(b) and 2(b) are without it). As for NGF (Figures 1(a) and 1(b)), data show great variability within the DS children group, which did not induce significant differences between DS and control groups (*p* = 0.201). Quite interestingly, a significant difference emerges in the BDNF serum levels between DS and control groups in the ANOVA for the Down Syndrome condition. Indeed, data indicate a potentiation in serum BDNF values (*p* = 0.04; Figure 2(b); *p* < 0.05 in the post hoc). Furthermore, no gender differences were observed for serum BDNF (Figure 2(a)).

### 3.3. Oxidative Stress Evaluation

**Figure 3:**
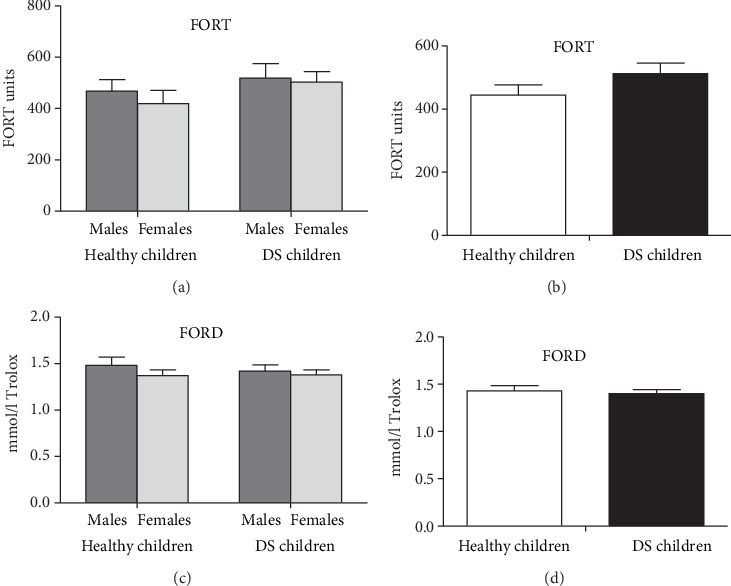
Free oxygen radicals test (FORT) and free oxygen radicals defense (FORD) levels in male and female DS prepubertal children and in the control group (the left panels represent the full interaction and the right panels are without the gender effect). The error bars indicate pooled standard error means (SEM) derived from appropriate error mean square in the ANOVA.

This study revealed no differences between DS children, and controls were found in FORD and FORT parameters, neither if analyzed for the gender parameter (Figure 3, left panels for the full interaction and the right panels without the gender effect).

### 3.4. Serum Levels of TNF-*α*, TGF-*β*, MCP-1, IL-1*α*, IL-2, IL-6, IL-10, and IL-12

**Figure 4:**
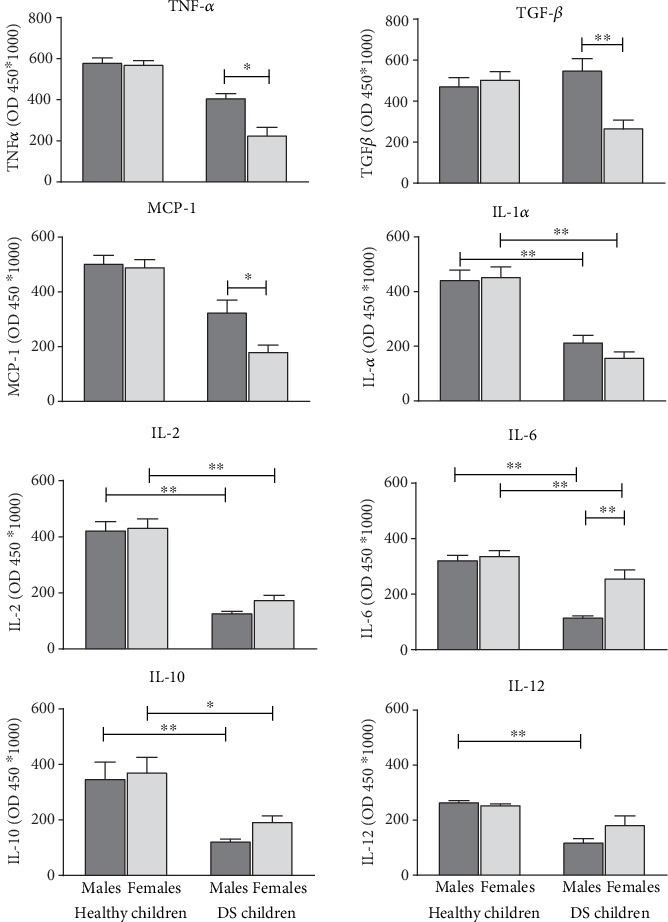
TNF-*α*, TGF-*β*, MCP-1, IL-1*α*, IL-2, IL-6, IL-10, and IL-12 serum levels in male and female DS prepubertal children and in the control group. The error bars indicate pooled standard error means (SEM) derived from appropriate error mean square in the ANOVA. The asterisks indicate significant differences between groups (^∗^*p* < 0.05); (^∗∗^*p* < 0.01).

Figure 4 shows the ANOVA data graphical expression of serum TNF-*α*, TGF-*β*, MCP-1, IL-1*α*, IL-2, IL-6, IL-10, and IL-12 of male and female children affected by Down Syndrome compared to healthy male and female children used as controls.

ANOVA clearly evidences differential expression of serum inflammatory cytokines between prepubertal DS children and controls. Indeed, low expression in DS prepubertal children, and in particular, in DS prepubertal female children were observed for TNF-*α*, TGF-*β*, and MCP-1 (*p* < 0.05 in the ANOVAs; *p* < 0.05 in post hoc between groups). Similar findings, but with decreased expression in DS prepubertal male children, were revealed for IL-6 and IL-12 (*p* < 0.01 in the ANOVAs; *p* < 0.01 in post hoc between groups). As for IL-1*α*, IL-2, and IL-10, data clearly disclose low serum expression of these cytokines in DS prepubertal children compared to controls with no differences due to gender (*p* < 0.05 in the ANOVAs; *p* < 0.05 in post hoc between groups).

## 4. Discussion

This is the first study investigating in prepubertal DS children the relationship between neurotrophins, oxidative stress, and neuroinflammatory markers in the serum. Indeed, we evidenced, although without NGF, FORD, and FORT changes, BDNF elevation and a marked reduction of TNF-*α*, TGF-*β*, MCP-1, IL-1*α*, IL-2, IL-6, IL-10, and IL-12. We also demonstrated for the first time different gender responses between males and females DS children in the analyzed cytokines.

As for oxidative stress in DS, we did not find changes in prepubertal DS children in the parameters of oxidation we investigated. However, some animal model and human studies have shown that the activity of the superoxide dismutase enzyme (SOD) is elevated in Down Syndrome [41, 42]. SOD converts oxygen radicals into hydrogen peroxide and water. Oxygen radicals produced in cells can be damaging to cellular structures, hence the important role of SOD. However, it has been hypothesized that if SOD activity increases disproportionately to enzymes responsible for the removal of hydrogen peroxide (e.g., glutathione peroxidase), the cells will suffer from peroxide damage. It has been speculated that the treatment of Down Syndrome neurons with free radical scavengers could prevent neuronal degeneration [31]. In fact, oxidative damage to neurons could lead to rapid brain aging similar to that of Alzheimer's disease. Quite interestingly, the DNA oxidation product 8-OHdG (8-Oxo-2′-deoxyguanosine (8-oxo-dG)) in adults with DS measured in saliva was found to be significantly higher than in the control groups [43]. 8-OHdG levels were also found to be higher in urine [44] and leukocytes [45] of persons with DS compared to controls. These findings suggest that oxidative DNA damage may underlie some of the clinical and premature aging features of DS.

For what concerns neurotrophins in DS, it has been previously shown that serum changes in the levels of both NGF and BDNF [46–48]. In the present study, we found BDNF elevation suggesting that high levels of circulating BDNF could protect DS patients from the clinical complications of atherosclerosis [46]. However, the striking drop in peripheral BDNF levels with age might predispose these patients to clinical manifestations of dementia in later life [46]. Quite interestingly, as previously reported [46], DS is considered a condition with a low risk of clinical atherosclerosis and cardiovascular disease during adult and later life. A potentiation of biomolecules with functional activities in endothelial cell activation [49, 50] should promote the risk of atherosclerosis in DS. However, since DS is considered an atheroma-free model [51] and clinical studies do not report any increase in the risk of cardiovascular disease in adult and elderly patients, DS individuals constitute a still unsolved biological/clinical paradox [47]. Among our cohort of DS prepubertal children, we did not disclose differences in NGF levels. However, a previous study did not reveal differences in serum NGF between prepubertal DS children and controls [48] while potentiation in NGF serum levels of DS children was reported in another investigation [47] demonstrating that the age parameter in DS children could be a confounding factor. Disrupted serum BDNF may correlate also with the elevated risk of dementia in DS individuals. Indeed, Alzheimer's disease is most likely universal in older individuals with Down Syndrome due to having three copies of the amyloid precursor protein gene, resulting in amyloid-beta plaque deposition [52, 53]. Older adults with Down Syndrome often present with cognitive decline: more than 80% may experience dementia by age 65 years. However, further investigation is required to better understand and disclose the biomarkers of the disorder progression and their relationship with symptom development during the presymptomatic period [52, 53].

DS individuals typically have a poor immune function and generally reach developmental milestones at a later age. They have an increased risk of a number of other health problems, including congenital heart defects, epilepsy, leukemia, thyroid and autoimmune diseases, and mental disorders. Indeed, DS persons may have a high frequency of infections, usually of the upper respiratory tract, characterized by increased severity and prolonged course of the disease, which are partially attributed to defects of the immune system. The abnormalities of the immune system associated with DS include mild to moderate T and B cell lymphopenia, with a marked decrease in naive lymphocytes, impaired mitogen-induced T cell proliferation, reduced specific antibody responses to immunizations, and defects of neutrophil chemotaxis. Limited evidence of genetic abnormalities secondary to trisomy of chromosome 21 and affecting the immune system is available, such as the potential consequences of gene overexpression [30]. Secondary immunodeficiency due to metabolic or nutritional factors in DS, particularly calcium, selenium, and zinc deficiency, has been also postulated [54]. However, the molecular mechanisms leading to the immune defects observed in DS individuals and the contribution of these immunological abnormalities remain largely unknown, especially in DS prepubertal children [55]. Children with Down Syndrome develop more infections, have an increased mortality from sepsis, and an increased incidence of chronic inflammatory conditions [56]. Cytokine dysregulation may underpin these clinical sequelae and raised proinflammatory biomarkers are a feature in adults with DS [56]. Changes in young and adult DS individuals' immune response is modulated by the levels of pro- and anti-inflammatory cytokines [57, 58] depending on many factors as the severity and extension of the trisomy 21, the presence of concomitant immunological disorders, infections, age, oxidative stress, congenital heart diseases, neurological impairments, and blood/nonblood cancers [59, 60]. In the sample of the present study of prepubertal DS children characterized by restricted recruitment criteria as no use of drugs or chemicals, concomitant inflammatory, endocrine and autoimmune disorders, or diagnosed cardiovascular pathologies, we found a significant decrease in the serum levels of TNF-*α*, TGF-*β*, MCP-1, IL-1*α*, IL-2, IL-6, IL-10, and IL-12. In addition, we disclosed for the first time different gender responses between males and females DS children in the analyzed serum cytokines. We do hypothesize that the low levels of the analyzed cytokines in the serum of DS prepubertal children could be due to a physiological delay and/or disrupted normal development of the individuals' immune system as previously reported for both DS subjects and DS animal models [61–63] with different cytokines presence between males and females prepubertal DS children. As for gender differences in DS children, available data show disparities between DS boys and girls mainly in major congenital heart defects [64, 65] and, as recently demonstrated, in emotional and behavioral problems associated with attention-deficit and hyperactivity disorders [66]. These data could be associated with the differences in serum cytokines analyzed in our study.

A further element of discussion, also related to the BDNF changes we found, is the relationship between these alterations in neuroinflammatory markers and the development of dementia in DS individuals. Actually, the specific chromosome 21 gene products and the complexity of the mechanisms they engender that give rise to the neuroinflammatory responses noted in fetal development of the DS brain and their potential as accelerators of Alzheimer neuropathogenesis in DS are crucial topics, particularly as they relate to the development and propagation of neuroinflammation, the consequences of which are recognized clinically and neuropathologically as dementia-like diseases [67].

We do believe that the strength of this study is that both controls and DS individuals underwent the same stressing conditions since they both came from the pediatrics section of a university hospital with similar blood collection rules and according to restricted subject recruitments' procedures (see methods, in particular for control individuals' enrollment). Furthermore, according to the exclusion criteria for the DS individuals' enrollment in the study, based on the university hospital standard analyses, we did not include children with anomalies in insulin-like growth factors (IGF-1) and thyroid hormones, quite common in DS children that could have biased the results on the neuroinflammatory markers. Of course, a limit of the study is the relatively low number of individuals enrolled in the experimentation but that depends on its strength (restricted enrollment rules). Since DS individuals display high variability in learning and memory abilities, another, but secondary, limit of the study is the lack of neurocognitive analyses to study changes in neurodevelopment and to correlate these findings also with the neuroinflammatory markers.

A future direction of this investigation by increasing the number of the enrolled subjects is the possibility to follow-up the patients over time to check whether or not early changes in neuroinflammatory markers could be associated with a premature onset of dementia-like diseases to early establish more efficacious treatment protocols.

The present investigation may represent a step forward in the attempt to unravel some biomolecular processes and responses underlying the DS immune system development and functionality. These results may be of interest also for studies in the fields of human genetic disorders in children.

## Figures and Tables

**Table 1:** Anamnestic and anthropometric data of the children or their parents included in the study. Data are expressed as means ± SE, as median, or as percentage.

	Down				Healthy			
Gender	Male		Female		Male		Female	
Number	5		4		10		11	
Age	6 ± 2.9		3.8 ± 5.6		7.13 ± 2.91		7.8 ± 3.4	
Weight (kg)	20.7 ± 9.6		15.8 ± 16.7		27.19 ± 18.6		27.25 ± 13.04	
Height (cm)	108.5 ± 23.1		86.3 ± 35.2		116.5 ± 27.5		126.5 ± 20.3	
BMI	17.39 ± 0.75		19.01 ± 2.97		19.55 ± 2.61		16.12 ± 0.92	
Feeding time (%)								
Breast milk	20		0		45.4		40	
Artificial milk	60		25		27.3		20	
Mixed milk	20		75		273		40	
Weaning (%)								
Before 5 months	0		0		0		30	
Between 5 and 6 months	60		75		100		30	
Over 6 months	40		25		0		40	
Birth (%)								
Normal	60		50		100		60	
Preterm (nonpathologic)	40		50		0		40	
Mode of delivery								
Vaginal	40		50		45.5		50	
Caesarean	60		50		54.5		50	
Age of parents to pregnancy								
Mother	36.75 ± 3.3		32.67 ± 8.14		32.6 ± 7.2		32.44 ± 8.62	
Father	44.75 ± 4.27		36.67 ± 3.79		35.9 ± 3.9		36.67 ± 8.47	
Educational level of parents (%)	Mother	Father	Mother	Father	Mother	Father	Mother	Father
No education	0	0	0	0	0	0	0	0
Primary school	0	0	0	0	0	0	0	0
Secondary school of I degree	0	60	0	0	36.4	27.25	20.0	30.0
Secondary school of II degree	100	40	100	60	27.2	4.5	50.0	30.0
University degree	0	0	0	40	36.4	27.25	30.0	40.0

## Data Availability

The biochemical and clinical data used to support the findings of this study are available from the corresponding author upon request.
